# The Crosstalk between Fat Homeostasis and Liver Regional Immunity in NAFLD

**DOI:** 10.1155/2019/3954890

**Published:** 2019-01-03

**Authors:** Minjuan Ma, Rui Duan, Hong Zhong, Tingming Liang, Li Guo

**Affiliations:** ^1^Jiangsu Key Laboratory for Molecular and Medical Biotechnology and College of Life Sciences, Nanjing Normal University, Nanjing, Jiangsu 210023, China; ^2^Department of Bioinformatics, School of Geographic and Biologic Information, Nanjing University of Posts and Telecommunications, Nanjing, Jiangsu 210023, China

## Abstract

The liver is well known as the center of glucose and lipid metabolism in the human body. It also functions as an immune organ. Previous studies have suggested that liver nonparenchymal cells are crucial in the progression of NAFLD. In recent years, NAFLD's threat to human health has been becoming a global issue. And by far, there is no effective treatment for NAFLD. Liver nonparenchymal cells are stimulated by lipid antigens, adipokines, or other factors, and secreted immune factors can alter the expression of key proteins such as SREBP-1c, ChREBP, and PPAR*γ* to regulate lipid metabolism, thus affecting the pathological process of NAFLD. Interestingly, some ncRNAs (including miRNAs and lncRNAs) participate in the pathological process of NAFLD by changing body fat homeostasis. And even some ncRNAs could regulate the activity of HSCs, thereby affecting the progression of inflammation and fibrosis in the course of NAFLD. In conclusion, immunotherapy could be an effective way to treat NAFLD.

## 1. Introduction

The liver, consisting of 80% hepatic parenchymal cells and 20% hepatic nonparenchymal cells, is the largest metabolism organ in the human body. It not only maintains the homeostasis of sugars and fats but also participates in immune regulation (innate immunity, adaptive immunity, and immune tolerance) [1–3] Hepatocytes are hepatic parenchymal cells, while the other 20% of nonparenchymal cells in the liver include liver sinusoidal endothelial cells (LSECs), natural killer (NK) cells, natural killer T (NKT) cells, Kupffer cells (KCs), hepatic stellate cells (HSCs), and dendritic cells (DCs). Hepatocytes, the parenchymal cells of the liver, are stimulated by a number of inflammatory factors, such as interleukin 6 (IL-6) and interleukin 1 beta (IL-1*β*), producing a large number of acute-phase proteins (APPs) that can kill foreign antigens and regulate immune responses [4]. KCs play an important role in the initial response to injury. When liver damage occurs, it can rapidly produce cytokines and chemokines, such as IL-1*β* and tumor necrosis factor *α* (TNF-*α*), resulting in the recruitment of other immune cells, such as monocytes. After entering the liver, the phenotype of monocytes changes, showing proinflammatory and profibrotic functions [5]. NK cells play an important role in liver host defense and immune balance by secreting cytokines, such as interferon gamma (IFN-*γ*) [6]. NKT cells serve as a bridge connecting innate immunity and adaptive immunity in the liver. They are activated by stimulation with lipid antigens, rapidly secrete anti-inflammatory or proinflammatory cytokines and chemokines, and participate in liver diseases [7]. A recent study shows that LSECs are serving as sentinel cells to detect microbial infection through pattern recognition receptor activation and as antigen (cross)-presenting cells [8]. Under normal physiological conditions, HSCs store liver lipids and 70% of the body's vitamin A. Conversely, after being stimulated by insulin, they can secrete type I collagen (Col-1) and connective tissue growth factor, causing inflammation and liver fibrosis [9]. Liver DCs internalize antigens and present them to surrounding lymphocytes as APCs and regulate T cells via the interleukin 10 (IL-10) mechanism [10, 11]. The hepatic metabolic homeostasis is maintained by the precise mechanism that each liver cell performs its own duties while maintaining crosstalk. Disorders of this mechanism can cause liver metabolic disorders and liver disease.

Liver disease (viral hepatitis, liver fibrosis, fatty liver, alcoholic liver, drug-induced liver damage and cirrhosis, and liver cancer), relating to liver immune cell disorders, is one of the reasons for high mortality in some countries and regions of the world [12–14]. Nonalcoholic fatty liver disease (NAFLD) is the most common cause of liver disease, the primary feature of which is simple steatosis caused by accumulation of liver triglycerides (TG), followed by nonalcoholic steatohepatitis (NASH) with inflammation, fibrosis, and liver damage, eventually developing into cirrhosis and hepatocellular carcinoma (HCC). Importantly, it is also closely related to obesity and type 2 diabetes, insulin resistance (IR), and other metabolic abnormalities [15, 16]. It has been reported that the prevalence of NAFLD is about 27%-34% in America [17], 25% in Europe [18], and 15-20% in Asia [19]. With economic development and changes in lifestyle, the upward trend of people's weights, especially in some high-income countries and regions, is causing global obesity and type 2 diabetes [20, 21]. Population-based studies showed that 2.6–9.6% of children have NAFLD, increasing up to 38–53% among obese children [22, 23]. Therefore, prevention and treatment of NAFLD is an imminent global issue.

NAFLD begins with excessive accumulation of TG in the liver. Studies have shown that approximately 60% of TG comes from adipose tissue free fatty acid pools, 26% from lipid de novo synthesis, and 15% from unhealthy diets [24]. Obesity and type 2 diabetes promote liver overload of glucose and insulin, which can activate carbohydrate response element binding protein (ChREBP) and sterol regulatory element binding protein-1c (SREBP-1c), respectively, thereby upregulating glycolytic enzymes and fatty acid synthase to promote hepatic lipogenesis [25]. Interestingly, IL-1*β* signaling mediates hepatocyte TG accumulation by driving the de novo lipogenic signaling pathway in obese mouse livers [26]. The main reason for the development of NASA from simple steatosis is abnormal release of adipokines and excessive accumulation of liver TG to activate hepatic nonparenchymal cells such as KCs, DCs, NK, and NKT. These cells can release proinflammatory factors including TNF-*α*, IL-6, and TGF-*β*, thereby leading to hepatitis, liver fibrosis, and liver damage [27].

Complex crosstalk between hepatocytes and other hepatocytes plays a decisive role in the pathogenesis of NAFLD. Surprisingly, some noncoding RNAs (ncRNAs) are involved in the pathological process of NAFLD and can even directly regulate certain pathways in liver nonparenchymal cells to influence the progression of NAFLD (Table 1). Therefore, this paper mainly clarifies the interaction between immune factors and ncRNA and protein in each stage of NAFLD, in order to provide potential targets for the treatment and prognosis of NAFLD.

## 2. Crosstalk between Hepatocytes, Hepatic Nonparenchymal Cells, and Adipocytes in the Pathological Process of NAFLD

### 2.1. Main Signal Pathways in Simple Hepatic Steatosis

Liver fat is mainly derived from dietary fatty acids, de novo lipogenesis, and adipose tissue. Hepatic steatosis occurs when fatty acid uptake, de novo lipogenesis, lipid secretion, or disposal via free fatty acid (FFA) oxidation are unbalanced [28].

Dietary fat is stored in fat tissue in the form of FFA to form an FFA pool. Obesity causes dysfunction of adipocytes and releases a large number of proinflammatory factors such as TNF-*α* [29], IL-6 [30], and leptin. This causes FFAs in the FFA pool to translocate to nonadipose tissues such as the liver and skeletal muscle [31]. At the same time, TNF-*α* inhibits the release of adiponectin by adipocytes, thereby increasing the uptake of hepatic FFAs and reducing hepatic fatty acid oxidation and TG output [32]. Excessive accumulation of FFAs causes IR [33], which can mobilize peripheral fat to form new FFAs. FFAs translocate to the liver, exacerbate liver TG accumulation, and ultimately cause hepatic steatosis [34].

De novo fatty acid synthesis is a complex reaction, and it is regulated by multiple factors [35]. This paper mainly describes the FFAs from de novo fatty acid synthesis through the pathways involved in the three proteins SREBP-1c, ChREBP, and PPAR*γ*. SREBP-1c, is an isoform of SREBPs (a family of TFs anchored in the ER membrane). It mainly regulates the synthesis of fatty acids [36]. ChREBP is a transcription factor that recognizes the carbohydrate response element (ChRE) in glycolysis and lipid synthesis gene promoter [37, 38]. The peroxisome proliferator-activated receptors (PPARs), transcription factors that regulate lipid metabolism and insulin sensitivity [39], consist of three isoforms: PPAR*γ*, PPAR*α*, and PPAR*δ* [40]. PPAR*γ* is highly expressed in adipocytes while it is also present in other hepatocytes, especially in resting HSCs [41].

IR can upregulate SREBP-1c, ChREBP, and PPAR*γ* to promote hepatic steatosis, which in turn can promote IR by SREBP-1c, ChREBP, and PPAR*γ*. (1) The proinflammatory factors TNF-*α* and IL-6 released by M1-type KCs (regulated by lipopolysaccharide and other factors) reach the hepatic portal vein with blood flow and form a functional complex with hepatocyte membrane receptors TNFR1 and IL-6R [42–44]. The complex formed by TNF-*α* and TNFR1 recruits upstream kinases such as nuclear factor-*κ*B- (NF-*κ*B-) inducible kinase (NIK) and transforming growth factor-*β*-activated kinase to activate NF-*κ*B, which upregulates IL-6 expression and cytokine signaling inhibitor-3 (SOCS-3) by binding to DNA [45]. IL-6 activates the Janus-activated kinase (JAK), which in turn is activated by phosphorylation of signal transduction and activator of transcription 3 (STAT3), which upregulates SOCS-3 [46]. SOCS-3 promotes IR by inducing the expression of SREBP-1c, which suppresses insulin receptor-mediated IRS1/2 synthesis [47]. (2) When IR causes severe extrahepatic injury, the liver tissue receives overloaded glucose and insulin. Insulin activates SREBP-1c [48]. SREBP-1c activates transcription of transcription factors involved in FFA metabolism, including the cluster of differentiation 36 (CD36) required for FFA uptake [49], FFA synthesis of required fatty acid synthase (FASN) and acetyl-CoA carboxylase (ACC), and TG synthesis-required glycerol-3-phosphate acyltransferase [50, 51]. High concentration of glucose induces the transfer of ChREBP from the cytoplasm to the nucleus; ChREBP in the nucleus upregulates FASN and ACC [52] and also induces glucose-6 phosphatase (G6P) and liver pyruvate kinase (LPK) expression to accelerate glycolysis and gluconeogenesis [53]. Interestingly, a recent study [54] found that activated ChREBP can directly target SREBP-1c and promote the accumulation of FFAs. (3) The large amount of FFAs released by adipose tissue activates liver PPAR*γ*, which upregulates fatty acid transporters (such as CD36), resulting in a significant increase in fatty acid uptake in hepatocytes. The fatty acid is then converted to fatty acyl CoA, which is ultimately esterified to TG. In addition, hepatic PPAR*γ* upregulates monoacylglycerol O-acyltransferase 1 (MGAT1) expression to promote TG accumulation [55]. PPAR*γ* also upregulates SREBP-1c expression to promote de novo synthesis of lipids [56].

### 2.2. Main Signal Pathways in NASH

The main pathological features of NASH are (1) oxidative stress (OS) [57], (2) systemic and hepatic inflammation [58], (3) fibrosis [59], and (4) hepatocyte apoptosis [60]. The appearance of these pathological features is mainly trigged by the accumulation of excessive FFAs in the liver to activate the signaling pathways that promote prooxidation, proinflammation, profibrosis, and proapoptosis [61].

FFAs are stored in lipid droplets in the form of TG, exported as very low-density lipoprotein (VLDL), and oxidized by mitochondrial *β*-oxidation. Excessive activation of mitochondrial *β*-oxidation and cytochrome P450 family 2 subfamily E member 1 (CYP2E1), a cytochrome that performs futile cycles with release of electrons into the cytosol, which destroy the electron transport chain, produces a large amount of reactive oxygen species (ROS), which ultimately leads to OS [62, 63]. TNF-*α* can effectively stimulate the production of mitochondrial ROS [64]. In order to reduce ROS, a large amount of mitochondrial uncoupling protein is produced to reduce superoxide anions, which unfortunately leads to a decrease in membrane potential, thereby limiting ATP production. When the ATP supply is insufficient, dying KCs release the proinflammatory factor interleukin 8 (IL-8). This recruits inflammatory cells into the liver [65].

FFAs activate proinflammatory signals through TLR4 and in a synergistic manner with LPS [66, 67]. Proinflammatory cells (including hepatocytes, KCs, and adipocytes) produce proinflammatory factors through NF-*κ*B activation [68]. LPS targets and activates TLR4 in hepatocytes and KCs, which upregulates NF-*κ*B transcription to produce a large number of proinflammatory factors including IL-*β*, TNF-*α*, IL-6, IL-8, and TGF-*β* [69]. KCs can also be activated by OS to promote the release of proinflammatory factors (IL-*β*, TNF-*α*, IL-6, IL-8, and TGF-*β*) [70]. The dysfunctional adipocytes release a large amount of the proinflammatory factors TNF-*α* and IL-6 [30]. The massive release of proinflammatory factors promotes the conversion of simple hepatic steatosis to NASH. At the same time, proinflammatory factors in turn promote IR, thus aggravating NAFLD. Adipocytes can release two adipokines, leptin and adiponectin, which are involved in inflammation and fibrosis to affect the pathological process of NASH [71]. Adiponectin reduces inflammation by stimulating KCs to secrete anti-inflammatory cytokines (such as IL-10), blocking the activation of NF-*κ*B [72], and inhibiting the release of TNF-*α*, IL-6, and chemokines, and antifibrosis by activating AMP-activated protein kinase (AMPK) in HSCs [73, 74]. Leptin accelerates the development of NASH by upregulating the expression of CD14 in KCs (enhancing the response of hepatocytes to low-dose LPS) [75]. It can also promote fibrosis by targeting KCs and sinusoidal endothelial cells, releasing a large amount of TGF-*β*, which promotes HSC production of collagen [76–79]. Unfortunately, low expression of adiponectin and high expression of leptin promote the development of inflammation and fibrosis in the NAFLD.

NF-*κ*B upregulates the expression of TNF-*α*, Fas ligand (FasL), and TGF-*β*, which are considered to be major factors in the response to apoptosis and fibrosis that drive NASH progression [80]. After TNF-*α* and FasL recognize and bind to receptor TNFR and Fas on hepatocytes, respectively, TNFR and Fas are activated, which triggers a series of apoptotic events. Homologous dimerization of activated TNFR and Fas induces the formation of the death-inducing signaling complex (DISC), which induces activation of caspases 8 and 10, and induction of caspase 3, 6, and 7, eventually leading to hepatocyte apoptosis [81, 82]. Upon activation of NKT cells by lipid antigens, CD40L expression is upregulated on the surface of NKT cells, which interacts with CD40 expressed on DCs, resulting in the production of IL-12 by DCs [83]. Increased liver IL-12 levels further stimulate liver NKT cells to enhance cytotoxicity by releasing perforin and granzymes and increasing the expression of FasL on the surface, which expresses binding to Fas receptors expressed on hepatocytes, leading to apoptosis [84]. Activation of NKT cells can also indirectly induce hepatocyte apoptosis by the release of cytokines, including IFN-*γ* and TNF-*α*. NKT-derived IFN-*γ* activates and recruits NK cells and DCs. Specifically, NK cell-induced transactivation of NK cells enhances cytolysis and production of NK cell IFN-*γ*, thereby further promoting hepatocyte apoptosis [85, 86].

The phosphorylation of SMAD2/3 is triggered by the interaction of TGF-*β* produced by KCs with specific receptors on the membrane of HSCs. Phosphorylated SMAD2/3 forms a complex with SMAD4 and is transferred to the nucleus to upregulate Col-1 in combination with target genes, thereby promoting fibrosis [87, 88].

## 3. ncRNAs Link the Fat Homeostasis and Immunity in the Progression of NAFLD

Noncoding RNA (ncRNA) refers to an RNA that does not encode any protein. ncRNA includes RNAs of known functions such as rRNA, tRNA, snRNA, snoRNA, and microRNA, as well as RNAs of unknown function. ncRNA plays an important role in the process of life. For example, ncRNAs can regulate mRNA stability and translation and ncRNAs affect protein stability and transport [89]. In the chapter, we will focus on the role of microRNA and lncRNA in the progression of NAFLD.

microRNAs (miRNAs) are endogenous, noncoding RNAs of about 20-24 nucleotides in length that can interact with messenger RNA and participate in posttranscriptional regulation of gene expression [90]. Studies have showed that the upregulation of microRNA-373 (miR-373) lowers its target gene AKT serine/threonine kinase 1 (AKT1) mRNA levels, resulting in suppression of the AKT-mTOR-S6K signaling pathway in hepatocytes and eventually weakening hepatic lipid abnormal deposition [91]. In zebrafish, the depletion of miR-7a increases the expression of Yin Yang 1 (YY1). YY1 induces the expression of C/EBP-*α* and PPAR*γ* by inhibiting the expression of CHOP-10, leading to the accumulation of FFAs and triglyceride, and finally causing NASH [92]. MiR-130a-3p promotes the apoptosis of HSCs and inhibits the production of collagen by inhibiting the TGF-*β*/SMAD signaling pathway, thereby improving the pathological process of the liver [93]. Overexpression of miR-26a improves NAFLD and reduces IL-17 expression by partially reducing IL-6 expression, indicating the immunomodulatory effects of the miR-26a-IL-6-IL-17 axis in the pathological process of NAFLD. However, the specific mechanism of immune regulation has not been elucidated [94]. MiR-146a-5p inhibits the activity and proliferation of HSCs by downregulating Wnt1 and Wnt5a; the amount of Col-1 is decreased, thereby inhibiting the occurrence of fibrosis in the progression of NAFLD [95]. Several of the above studies indicate that miRNAs affect a particular signaling pathway by targeting their specific target genes, thereby regulating lipid-metabolizing proteins, as well as hepatic nonparenchymal cells involved in NAFLD progression. So, miRNAs may serve as new targets for NAFLD.

Long-chain noncoding RNAs are noncoding RNAs with a length of more than 200 nt, which have important functions in transcriptional silencing, transcriptional activation, chromosome modification, and nuclear transport [96, 97]. As transcription factors, lncRNAs play an important role in normal physiological and pathological processes [98]. In 2015, a study reported that Berberine (BBR) can upregulate the expression of lncRNA MRAK052686 and its target genes *Nrf2* and *Eif2ak2* in a high-fat diet- (HFD-) induced steatotic animal model, thereby inhibiting the PERK (PKR-like ER-kinase) pathway in ER stress, indicating that lncRNA MRAK052686 plays a role in NAFLD by affecting ER stress. This study opened the prelude to the study of the role of lncRNAs in NAFLD [99]. lncRNA SRA inhibits the transcription of forkhead box protein O1 (FoxO1) through an insulin-independent pathway in hepatocytes, thereby reducing the expression of the downstream gene adipose triglyceride lipase (ATGL) and subsequently lowering the FFA *β*-oxidation of hepatocytes, resulting in hepatic steatosis [100]. Some lncRNAs are involved in immune regulation by regulating nonparenchymal liver cells. Studies have found that in activated HSCs, lncRNA MALAT1 expression is upregulated and upregulates its target C-X-C motif chemokine ligand 5 (CXCL5), thereby promoting the development of inflammation and fibrosis in NASH [101]. Correspondingly, the study found that lncRNA NONRATT013819.2 was involved in ECM-related pathways to mediate activation of HSCs by upregulating the expression of lysyl oxidase (Lox) [102]. LncRNAs are not only involved in the development of NAFLD, but also related to the occurrence of HCC [103]. Most importantly, lncRNAs are involved in immune regulation [104], indicating that lncRNAs can be used as a novel target for liver immunotherapy.

## 4. Conclusions and Immune Treatment Strategies in NAFLD

**Figure 1:**
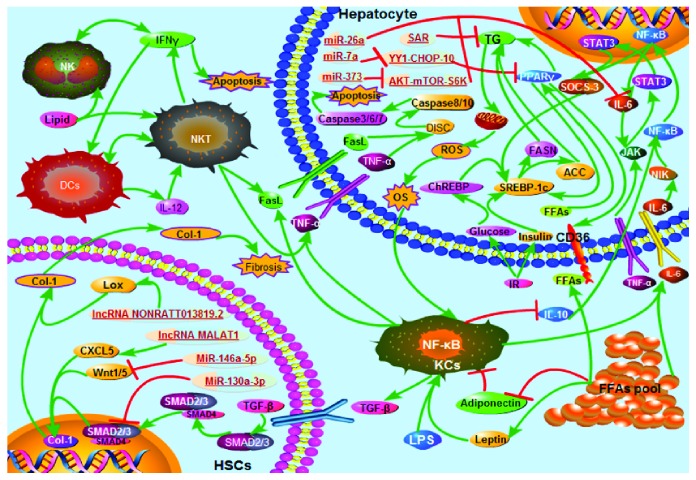
Crosstalk between hepatocytes, hepatic nonparenchymal cells, and ncRNA during the pathology of NAFLD. (1) Hepatic steatosis. Obesity and LPS from the intestine promote the release of proinflammatory cytokines TNF-*α* and IL-6 from KCs and adipocytes, which interact with ligands on the hepatocyte membrane. TNF-*α* activates SREBP-1c via the JAK/STAT3/SOCS-3 pathway, which activates FASN and ACC to promote hepatic TG accumulation. IL-6 upregulates IL-6 and SOCS-3 by NIK/NF-*κ*B. The FFAs released from the FFA pool in the adipocytes directly activate PPAR*γ*, which upregulates SREBP-1c to promote TG accumulation through other pathways. IR causes liver overload of glucose and insulin, and glucose activates ChREBP, which not only activates FASN and ACC but also activates SREBP-1c. Eventually, TG overload in hepatocytes causes hepatic steatosis. (2) NASH. Deregulation of adipocytes causes an increase in leptin levels, but adiponectin levels decrease. Decreased adiponectin causes a decrease in the release of the anti-inflammatory factor IL-10 from KCs, thereby promoting inflammation. TG overload causes excessive mitochondrial *β*-oxidation, which produces large amounts of ROS, which causes OS, causing KCs to release large amounts of proinflammatory factors (TNF-*α*, IL-6, and TGF-*β*) and FasL. Upon recognition of TNF-*α* and FasL with ligands on the hepatocyte membrane, they induce the formation of a death-inducing signaling complex (DISC), which induces hepatocyte apoptosis via the caspase pathway. Leptin can promote the release of TGF-*β* by KCs. After binding of TGF-*β* to ligand recognition on HSCs, liver fibrosis is promoted through the SMAD/Col-1 pathway. Lipids can induce NK and NKT release of apoptotic factors IFN-*γ*, FasL, and TNF-*α*. Activated NKT activates DCs, which produce IL-12 that stimulates NKT and ultimately aggravates inflammation and apoptosis. microRNA-373 (miR-373) reduces inflammation by inhibiting the AKT-mTOR-S6K signaling pathway, which inhibits IL-6 production; miR-7a upregulates YY1, which induces the expression of PPAR*γ* by inhibiting the expression of CHOP-10, leading to the accumulation of FFAs and triglyceride, and finally causing NASH; MiR-130a-3p promotes the apoptosis of HSCs and inhibits the production of collagen by inhibiting the TGF-*β*/SMAD signaling pathway, thereby improving the pathological process of the liver; miR-26a improves NAFLD by partially reducing IL-6 expression; MiR-146a-5p inhibits the activity and proliferation of HSCs by downregulating Wnt1 and Wnt5a, and the amount of Col-1 is decreased, thereby inhibiting the occurrence of fibrosis in the progression of NAFLD; lncRNA SRA aggravates hepatic steatosis by reducing mitochondrial *β*-oxidation. lncRNA MALAT1 upregulates its target C-X-C motif chemokine ligand 5 (CXCL5), promoting the development of inflammation and fibrosis in NASH; and lncRNA NONRATT013819.2 promotes fibrosis by upregulating the expression of lysyl oxidase (Lox).

Nowadays, the number of people suffering from NAFLD is increasing rapidly [105]. However, because of the complicated pathogenic mechanism behind NAFLD, there is currently no effective treatment for NAFLD. During the NAFLD process, hepatocytes, hepatic nonparenchymal cells, and adipocytes crosstalk, releasing factors involved in hepatic steatosis, nonalcoholic steatohepatitis, fibrosis, and even HCC [106]. Accumulation of fatty acids causes liver steatosis [107], endoplasmic reticulum resistance, and reactive oxygen species to promote steatosis and development into NASH [108]. By far, the existing methods for treating NAFLD include improving diet, strengthening exercise, changing lifestyles, and performing weight loss surgery, all with the purpose of reducing the accumulation of liver fat in patients [109–114]. Studies on NAFLD have reported that there is a complicated relationship between liver immunity and fat homeostasis [115–117], while there is evidence that ncRNAs can interact with nonparenchymal liver cells and affect NAFLD [118]. Many functions of ncRNAs are not to be noted. Liver nonparenchymal cells play a crucial role in the maintenance of liver morphological structure and function (Figure 1). Although there are no reports of immunotherapy of NAFLD associated with fatty acid metabolism, future researches on the relationship between ncRNAs and NAFLD, between liver nonparenchymal cells and NAFLD, or the effect of interaction between ncRNAs and liver nonparenchymal cells on NAFLD, and liver immunotherapy for NAFLD could be a breakthrough point in addressing current treatment deficiencies. Therefore, searching the target of NAFLD immunotherapy could be a new direction in the research for the treatment of NAFLD.

## Figures and Tables

**Table 1:** ncRNAs link the fat homeostasis and immunity in the pathological process of NAFLD.

Noncoding RNAs	Target genes	Signaling pathways	References
MiR-373	*AKT1*	AKT–mTOR-S6K	[91]
MiR-7a	*YY1*	YY1-CHOP-10-C/EBP-*α*/PPAR*γ*	[92]
MiR-130a-3p	*TGFBR1/TGFBR2*	GF-*β*/SMAD	[93]
MiR-26a	*IL-6* mRNA	miR-26a-IL-6-IL-17 axis	[94]
MiR-146a-5p	*Wnt1* and *Wnt5a*	Wnt	[95]
LncRNA MRAK052686	*Nrf2* and *Eif2ak2*	PERK	[99]
LncRNA SRA	*FoxO1*	FFA *β*-oxidation	[100]
LncRNA MALAT1	*CXCL5*	Extracellular space	[101]
LncRNA NONRATT013819.2	*Lox*	ECM-related pathways	[102]

## Abbreviations

NK: Natural killer cells
NKT: Natural killer T cells
LSECs: Liver sinusoidal endothelial cells
IL-6: Interleukin 6
IL-1*β*: Interleukin 1 beta
APPs: Acute-phase proteins
KCs: Kupffer cells
TNF-*α*: Tumor necrosis factor *α*
IFN-*γ*: Interferon gamma
HSCs: Hepatic stellate cells
NAFLD: Nonalcoholic fatty liver disease
Col-1: Type I collagen
NASH: Nonalcoholic steatohepatitis
IR: Insulin resistance
SphK1: Sphingosine kinase 1
FFAs: Free fatty acids
SREBP-1c: Sterol regulatory element binding protein-1c
ChREBP: Carbohydrate response element binding protein
PPAR*γ*: Peroxisome proliferator-activated receptor-*γ*
SOCS-3: Cytokine signaling-3
G6p: Glucose-6-phosphatase
LPK: L-type pyruvate kinase
OS: Oxidative stress
VLDL: Low-density lipoprotein
CYP2E1: Cytochrome P450 family 2 subfamily E member 1
FasL: Fas ligand
DISC: Death-inducing signaling complex
FASN: Fatty acid synthase
ACC: Acetyl-CoA carboxylase
IL-10: Interleukin 10
HCC: Hepatocellular carcinoma
ROS: Reactive oxygen species production
JNK: c-Jun N-terminal kinase
MGAT1: Monoacylglycerol O-acyltransferase 1
LPS: Lipopolysaccharide
AMPK: AMP-activated protein kinase
ncRNA: Noncoding RNA
AKT1: AKT serine/threonine kinase 1
YY1: Yin Yang 1.
